# Metabolomics analysis unveils important changes involved in the salt tolerance of *Salicornia europaea*

**DOI:** 10.3389/fpls.2022.1097076

**Published:** 2023-01-20

**Authors:** Huirong Duan, Richard John Tiika, Fuping Tian, Yuan Lu, Qian Zhang, Yu Hu, Guangxin Cui, Hongshan Yang

**Affiliations:** ^1^ Lanzhou Institute of Husbandry and Pharmaceutical Science, Chinese Academy of Agricultural Sciences, Lanzhou, China; ^2^ College of Forestry, Gansu Agricultural University, Lanzhou, China

**Keywords:** *Salicornia europaea*, salt tolerance, metabolomics, flavonoids, phenolic acids

## Abstract

*Salicornia europaea* is one of the world’s salt-tolerant plant species and is recognized as a model plant for studying the metabolism and molecular mechanisms of halophytes under salinity. To investigate the metabolic responses to salinity stress in *S. europaea*, this study performed a widely targeted metabolomic analysis after analyzing the physiological characteristics of plants exposed to various NaCl treatments. *S. europaea* exhibited excellent salt tolerance and could withstand extremely high NaCl concentrations, while lower NaCl conditions (50 and 100 mM) significantly promoted growth by increasing tissue succulence and maintaining a relatively stable K^+^ concentration. A total of 552 metabolites were detected, 500 of which were differently accumulated, mainly consisting of lipids, organic acids, saccharides, alcohols, amino acids, flavonoids, phenolic acids, and alkaloids. Sucrose, glucose, p-proline, quercetin and its derivatives, and kaempferol derivatives represented core metabolites that are responsive to salinity stress. Glycolysis, flavone and flavonol biosynthesis, and phenylpropanoid biosynthesis were considered as the most important pathways responsible for salt stress response by increasing the osmotic tolerance and antioxidant activities. The high accumulation of some saccharides, flavonoids, and phenolic acids under 50 mM NaCl compared with 300 mM NaCl might contribute to the improved salt tolerance under the 50 mM NaCl treatment. Furthermore, quercetin, quercetin derivatives, and kaempferol derivatives showed varied change patterns in the roots and shoots, while coumaric, caffeic, and ferulic acids increased significantly in the roots, implying that the coping strategies in the shoots and roots varied under salinity stress. These findings lay the foundation for further analysis of the mechanism underlying the response of *S. europaea* to salinity.

## 1 Introduction

Land salinization is one of the most serious abiotic stresses affecting modern agriculture worldwide, which limits the crop productivity and spatial distribution of plants (Morton et al., 2019; Gong et al., 2020). Nearly 10% of the land surface and 50% of irrigated land in the world are affected by salinized soils (Zhang et al., 2020a). The rising problem of global soil salinization and the serious losses in crop production call for a better understanding of the key mechanisms of salt tolerance in crops (Shabala and Mackay, 2011; Duan et al., 2015). An effective way of obtaining such information comes from studying halophytes. Halophytes possess remarkable abilities to tolerate and even benefit from the saline environment that may kill most other plant species (Flower et al., 2010; Shabala, 2013). Over the past few decades, although many important mechanisms for salt tolerance in halophytes have been intensively studied, breeding of crop plants with salt tolerance has not been very successful to date (Lu et al., 2020; Wang et al., 2020). Salt tolerance of plants is an intricate multigenic trait exhibiting heterosis, dominance, and additive effects and is also physiological omnifariously under the regulation of multiple tissue- and age-specific components (Shabala and Cuin, 2008). Therefore, salt tolerance of halophytes will be reflected by numerous subtraits, and comprehensive and systematic analyses focusing on salt tolerance should be conducted extensively.

The changes of metabolites reflect how an organism systemically and directly adapts to environmental changes (Weckwerth, 2003; Bundy et al., 2009). As a developing and promising methodology, metabolomics can analyze the metabolite changes qualitatively and quantitatively in organisms, thus revealing the relationship between plant species and environment and also showing the connection among phenotypes, metabolic networks, metabolic regulation, function, and plant growth (Schauer and Fernie, 2006; Töpfer et al., 2015). Plant metabolism is affected under salt stress, and plants need to adjust their metabolic levels to maintain basic metabolism and reach new homeostasis (Shulaev et al., 2008; Arbona et al., 2013). Consequently, metabolomics is the suitable tool for investigating plants’ responses to salt stress. The metabolic responses of some plants to salt stress have already been examined, for example, *Suaeda salsa*, *Atriplex halimus*, and *Hordeum vulgare*, they provided new insights into the physiological mechanism of salt tolerance and nutritional value in halophytic species (Nemat Alla et al., 2012; Shelden et al., 2016; Li and Song, 2019).

*Salicornia europaea*, belonging to Amaranthaceae, is an annual euhalophyte widely distributed in coastal and inland salt marshes (Nie et al., 2015; Lv et al., 2020). As one of the most salt-tolerant plant species worldwide, the growth of *S. europaea* can be stimulated by 200 to 400 mM NaCl, and it can even withstand more than 1,000 mM NaCl (Lv et al., 2015). Thus, *S. europaea* is recognized as a model plant for studying the metabolism and molecular mechanisms of halophytes under salinity (Feng et al., 2015). A series of studies have been conducted in *S. europaea* under different salt treatments, which indicated that metabolic responses were caused under high salinity, including glycine betaine, proline, trigonelline, D-(+)-glucose, 2-propenyl (sinigrin), fructose-1-phosphate, and so on in the seedlings of *S. europaea* (Guy et al., 1984; Momonoki et al., 1994; Moghaieb et al., 2004; Wang et al., 2021). However, comprehensive studies on the metabolomics of *S. europaea* in a saline environment are limited, and how *S. europaea* adapts to the saline habitat by regulating the change of metabolites needs to be further deciphered.

In the present study, the effects on the growth and ion accumulation of *S. europaea* under salt treatments were compared. Moreover, the changes in the metabolic profiles of *S. europaea* under salinity were analyzed by widely targeted metabolomics. Our results comprehensively depict the characteristics of *S. europaea* metabolic response under salinity and provide a vigorous foundation for further understanding of the salt tolerance mechanisms and evaluating the nutritional value in *S. europaea*.

## 2 Materials and methods

### 2.1 Plant material and growth conditions

Seeds of *S. europaea* were collected from Liangcao Village of Jingtai County (37°21′2″, 104°5′28″), Gansu Province, China. Healthy seeds were rinsed with distilled water for three to five times after sterilization with 2% NaClO for 5 min. Thereafter, the seeds were germinated on a filter paper under 26°C in the dark. Uniform seedlings with a 2 to 3 cm radicle were transplanted into plastic pots (5 cm × 5 cm × 5 cm; one seedling/pot) containing sterilized sand. These seedlings were grown in an artificial climate chamber with a day/night temperature of 26°C/23°C. The daily photoperiod was 16/8 h (light/dark), with a light flux density of 600 μmol/m^2^·s, and the relative humidity was 65%. The seedlings were divided into 10 groups and were watered with modified 1/2 strength Hoagland nutrient solutions (Tiika et al., 2021) supplemented with different NaCl concentrations (0, 50, 100, 200, 300, 400, 500, 600, 700, and 800 mM), respectively.

### 2.2 Growth index measurements

The seedlings were harvested 30 days after the imposition of different NaCl concentrations (0–400 mM) and then rinsed in distilled water. Four independent biological replicates of shoot and root samples were collected for this analysis. The fresh weight (FW) of separated root and shoot tissue, root length, and shoot height were measured. Thereafter, the samples were dried in an oven at 105°C for 10 mins before adjusting to 80°C for 72 h, and the dry weight (DW) of root and shoot tissue was determined. The degree of succulence was calculated by the following equation: succulence degree = FW of shoots / DW of shoots (Qi et al., 2009).

### 2.3 Measurement for Na^+^ and K^+^ concentrations in plants

Uniform seedlings were collected for the measurement of Na^+^ and K^+^ concentrations after treatment with additional NaCl for 30 days. The roots of the seedlings were rinsed twice (once for 4 min) in ice-cold 20 mM CaCl_2_ to exchange cell wall-bound Na^+^, and then the shoots were washed in deionized water to remove surface salts (Ma et al., 2017). Four independent biological replicates of shoot and root samples were harvested. Then, the tissues were dried at 80°C for 72 h to obtain dry weights. Na^+^ and K^+^ were extracted from the dried root and shoot tissues in 100 mM acetic acid at 90°C for 2 h, and the ions were assayed using atomic absorption spectrophotometry (2655-00, Cole-Parmer Instrument Co., Vernon Hills, USA).

### 2.4 Sample preparation and extraction

Seedlings treated with 0 mM (CK), 50 mM (L), and 300 mM (H) NaCl for 30 days were rinsed in distilled water and then collected for widely targeted metabolic profiling analyses. Three independent biological replicates of shoot and root samples were collected under each NaCl condition. The root and shoot were freeze-dried and then crushed to powder using a mixer mill (MM 400, Retsch) with a zirconia bead at 30 Hz for 1.5 min (Li and Song, 2019). Furthermore, 100 mg powder was dissolved with 0.6 ml of 70% aqueous methanol, vortexing for 30 s every 30 min for six times, and was extracted overnight at 4°C (Sun et al., 2020). Following centrifugation at 10,000 *g* for 10 min, the extracts were absorbed and filtrated with a 0.22 μm pore size Nylon Syringe Filter (SCAA-104, anpel, Shanghai, China) before the UPLC–MS/MS analysis. Quality control (QC) samples were prepared by mixing sample extracts.

### 2.5 Data acquisition and quality control

The sample extracts were analyzed using an UPLC–ESI–MS/MS system (UPLC, Shim-pack UFLC SHIMADZU CBM30A system; MS, Applied Biosystems 4500 Q TRAP) from Metvare Biotechnology Co., Ltd., Wuhan, China. The analytical conditions were set according to the report of Li and Song (2019). To monitor the technical reproducibility, one QC sample was inserted into each of the 10 detected samples during the stability evaluation of the analysis conditions (Yi et al., 2021). Qualitative annotations of the metabolites were obtained from the Metware database together with the public databases. Quantifications of metabolites were conducted based on the information of retention time and peak pattern of metabolites and normalized by the R program (www.r-project.org/) (Xiao et al., 2021). Then, hierarchical cluster analysis was performed on the accumulation patterns of metabolites and visualized with a heat map.

### 2.6 Analysis of different metabolites

Multivariate statistical analysis combined with orthogonal partial least squares discriminant analysis and principal component analysis was conducted to analyze and verify the differences and reliability of metabolites in the samples. Different metabolites were screened by the thresholds with variable importance in projection (VIP) ≥ 1 and fold change (FC) ≥ 2 or ≤ 0.5. Then, the different metabolites were mapped to the Kyoto Encyclopedia of Genes and Genomes (KEGG) database and used in the significant enrichment analysis and major enriched pathways. To further analyze the change trend of relative content of metabolites, the relative contents of different metabolites were normalized and centralized, and then K-means clustering analysis was performed.

### 2.7 Statistical analysis

The physiological values were performed by ANOVA using SPSS statistical software (Version 16.0, SPSS Inc., Chicago, IL, USA). Duncan’s multiple range tests were used to detect differences among means at a significance level of *p* < 0.05.

## 3 Results

### 3.1 Growth performance of *S. europaea* under salt stress

**Figure 1:**
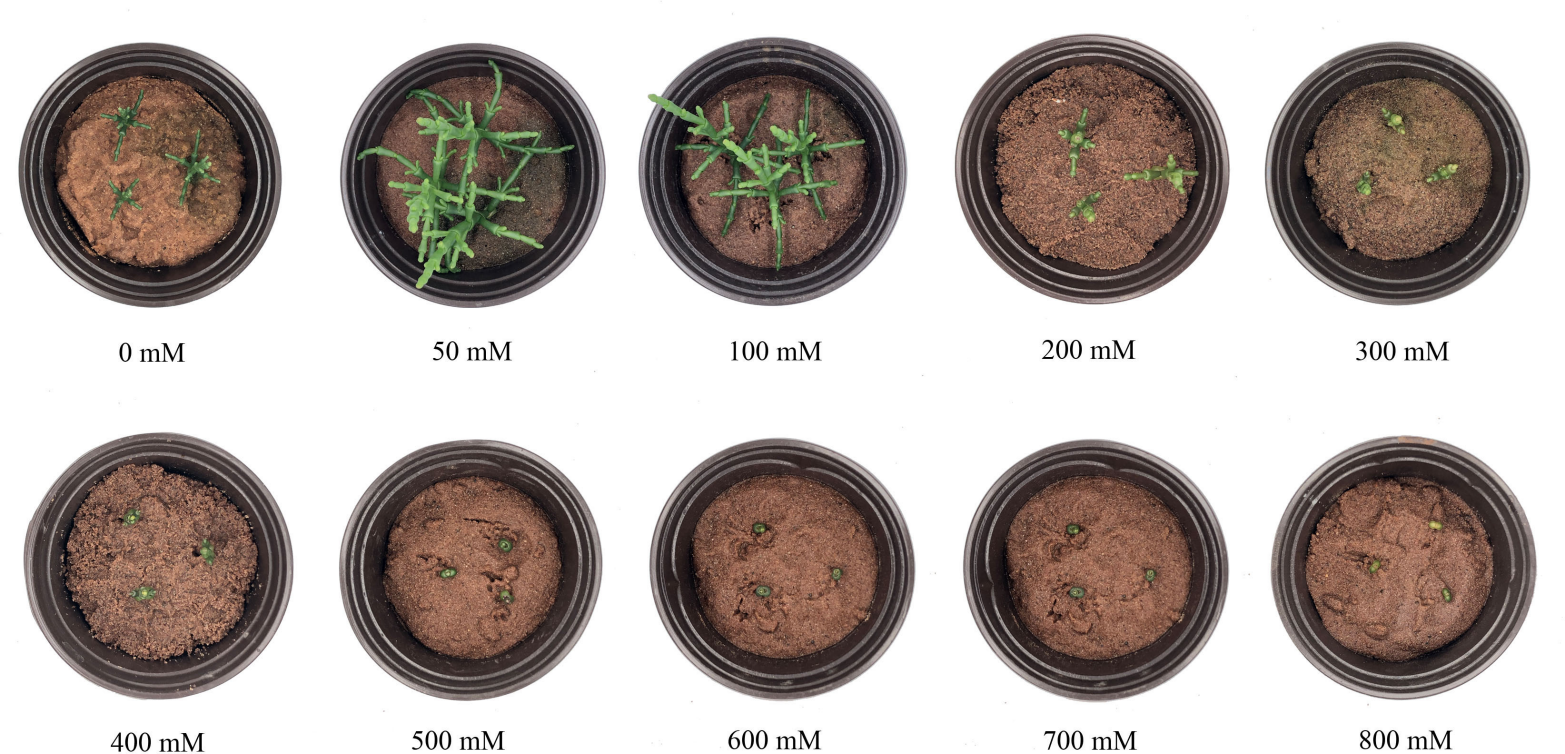
Growth performance of *Salicornia europaea* seedlings exposed to different levels of salt stress for 30 days.

**Figure 2:**
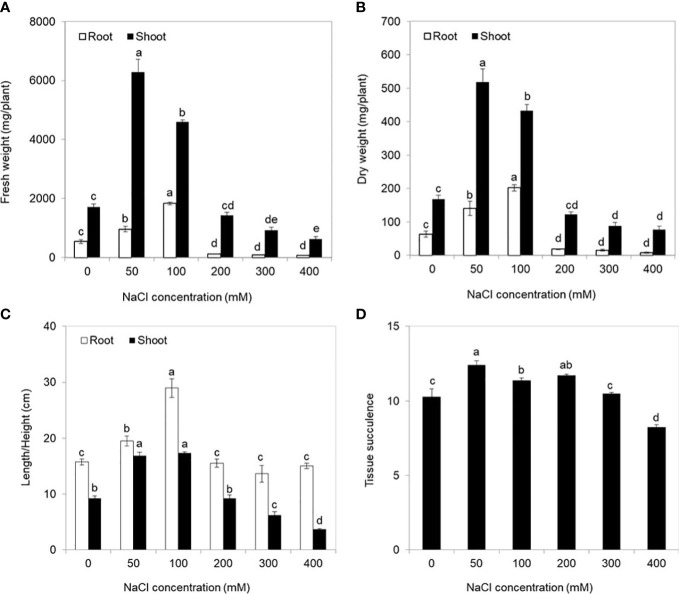
Physiological indices of *Salicornia europaea* seedlings exposed to different levels of salt stress for 30 days. **(A)** Fresh weight, **(B)** dry weight, **(C)** length/height, and **(D)** tissue succulence. Values are the means ± standard errors (SE) (*n* = 4), and bars indicate SE. Significant differences between columns in the roots and shoots are indicated by different lowercase letters (Duncan’s test, *p* < 0.05).

There were obvious differences in growth performances of *S. europaea* under different NaCl conditions (**Figure 1**). Severe salt stresses (≥ 300 mM NaCl) significantly reduced the growth of *S. europaea*, but *S. europaea* was still alive even under 800 mM NaCl. The fresh weights of shoots increased evidently and peaked at 50 and 100 mM NaCl, which were approximately 3.7- and 3.4-fold higher, respectively, than that under control condition (0 mM NaCl), and then reduced significantly (**Figure 2A**). The fresh weights of roots increased quickly with NaCl increasing and peaked at 100 mM NaCl, then decreased. The dry weights of shoots and roots exhibited similar change patterns (**Figure 2B**). Compared with the control, 50 and 100 mM NaCl promoted shoot height and root length significantly, and relative higher NaCl conditions (200–400 mM) reduced the shoot height evidently (**Figure 2C**). Besides, the tissue succulence of *S. europaea* increased firstly, and peaked at 50 mM NaCl, then decreased gradually with increasing NaCl concentrations (**Figure 2D**).

### 3.2 Ion accumulation of *S. europaea* under salt stress

**Figure 3:**
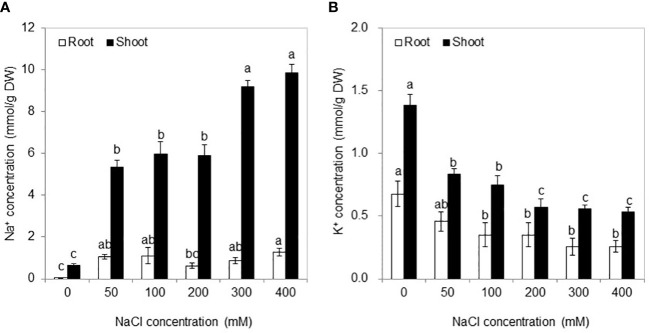
Na^+^ **(A)** and K^+^ **(B)** concentrations in the roots and shoots under different NaCl concentrations for 30 days. Values are means ± standard errors (SE) (*n* = 4), and bars indicate SE. Significant differences between columns in the roots and shoots are indicated by different lowercase letters (Duncan’s test, *p* < 0.05).

Na^+^ and K^+^ were primarily distributed in the shoots after NaCl treatments as well as in the absence of salt (0 mM NaCl) (**Figure 3**). With NaCl application, the Na^+^ concentration in roots and shoots was significantly higher than that in the control, and no significant difference was found among the 50–200 mM NaCl conditions and or between 300 and 400 mM NaCl conditions. Conversely, with the increase of salt stress, the K^+^ concentration in both the shoots and roots exhibited declining levels and then remained stable from the 200 mM NaCl condition to the 400 mM NaCl condition.

### 3.3 Metabolite statistics of *S. europaea*

**Figure 4:**
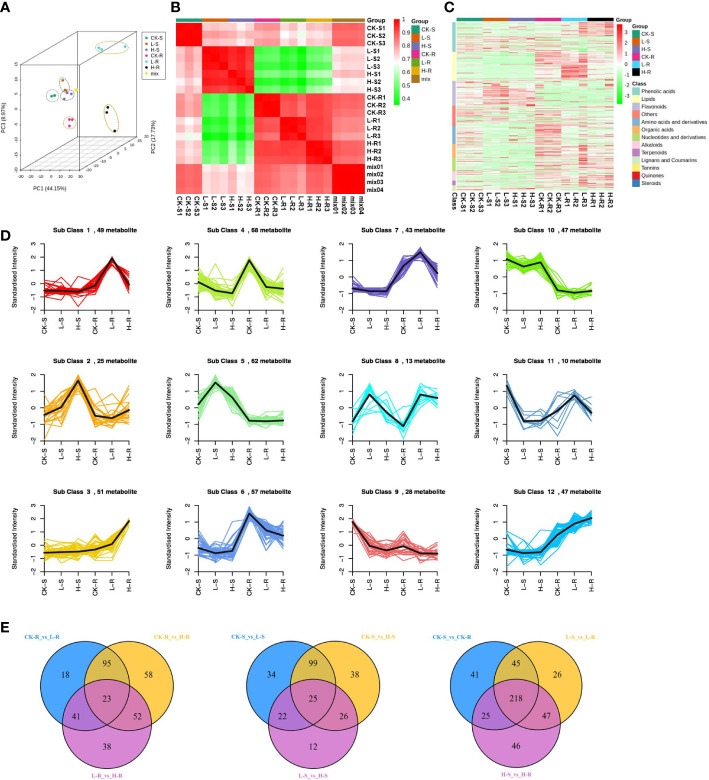
Dynamic metabolome of *Salicornia europaea* seedlings under different NaCl conditions. **(A)** Principal component analysis 3D plot of metabolomics data from three different NaCl treatments of the roots (R) and shoots (S). CK, 0 mM NaCl; L, 50 mM NaCl; H, 300 mM NaCl. “Mix” means the balanced mixture of all the samples (quality control). **(B)** Pearson correlation analysis of all the samples. “Mix” means the balanced mixture of all the samples (quality control). **(C)** Heat map of hierarchical clustering analysis of all detected metabolites in the roots and shoots among three different NaCl concentrations. Each sample is represented by a column, and each metabolite is represented by a row. **(D)** K-means cluster analysis of metabolites according to their variation tendencies. **(E)** Venn diagrams of different metabolites in multiple pairwise comparisons.

To investigate the metabolic changes of *S. europaea* adapted to different salt conditions, we carried out three different NaCl concentrations of *S. europaea* for extensive metabolic analysis using the UPLC-MS-based metabolomics approach, including 0, 50 mM NaCl (positively promoted concentration) and 300 mM NaCl (inflection point of inhibition concentration). As shown in **Figures 4A, B**, the three replicates of shoot and root from each treatment exhibited similar PC scores and were clustered together by Pearson’s correlation analysis, revealing small variations among replicates. The root samples from different NaCl concentrations presented more discrete distribution than the shoot samples. After identification and analysis, a total of 552 metabolites were detected, and assigned to different classes, including 96 phenolic acids, 95 lipids, 81 flavonoids, 58 amino acids and their derivatives, 45 organic acids, 44 nucleotides and their derivatives, 30 alkaloids, 15 terpenoids, 12 lignans and coumarins, 4 tannins, 3 quinones, 3 steroids, and 66 others (**Figure 4C**; **Supplementary Table S1**).

### 3.4 Analysis and enrichment of the different metabolites of *S. europaea*

A total of 500 different metabolites were screened among the different NaCl conditions based on a VIP value ≥ 1 and FC ≥ 2 or ≤ 0.5. Based on the K-means analysis result, 12 classes that exhibited distinct clustering of different metabolite variations were separated, and class 4 contained the most different metabolites with a total of 68 (**Figure 4D**). In order to evaluate the significant variations of metabolites among different tissues and NaCl concentrations, comparisons were conducted among different groups of CK-R *versus* (*vs*.) L-R, L-R *vs*. H-R, CK-S *vs*. L-S, L-S *vs*. H-S, CK-R *vs*. CK-S, L-R *vs*. L-S, and H-R *vs*. H-S (**Table 1**). In the same plant tissue, the highest number of different metabolites was found in CK-R *vs*. H-R, including 112 upregulated and 116 downregulated metabolites, suggesting that the 300 mM NaCl condition significantly influenced metabolite accumulation in the roots. Under the same treatment, more abundant different metabolites accumulated in the roots of *S. europaea*, except for flavonoids which were more accumulated in the shoots. After searching the top 20 different metabolites in CK-S *vs*. CK-R, L-S *vs*. L-R, and H-S *vs*. H-R, lipids, flavonoids, and phenolic acids were the top three types of metabolites that accumulated differently in the shoots and roots. Additionally, venn diagrams were constructed to analyze the common different metabolites among all the groups compared. A total of 23 different metabolites were common in CK-R *vs*. L-R, CK-R *vs*. H-R, and L-R *vs*. H-R; 25 different metabolites were common in CK-S *vs*. L-S, CK-S *vs*. H-S, and L-S *vs*. H-S; and 218 different metabolites were common in CK-S *vs*. CK-R, L-S *vs*. L-R, and H-S *vs*. H-R (**Figure 4E**).

**Table 1:** Comparison of different metabolites in the shoots and roots of *Salicornia europaea* under salt stress.

Group class	CK-R *vs* L-R		L-R *vs* H-R		CK-R *vs* H-R		CK-S *vs* L-S		L-S *vs* H-S		CK-S *vs* H-S		CK-S *vs* CK-R		L-S *vs* L-R		H-S *vs* H-R	
	Upregulated	Downregulated	Upregulated	Downregulated	Upregulated	Downregulated	Upregulated	Downregulated	Upregulated	Downregulated	Upregulated	Downregulated	Upregulated	Downregulated	Upregulated	Downregulated	Upregulated	Downregulated
Phenolic acids	19	15	13	6	31	16	17	8	3	12	16	12	32	26	31	24	37	25
Lipids	33	6	2	41	5	17	0	23	5	2	1	16	64	9	77	7	63	6
Flavonoids	3	8	23	3	16	8	24	4	5	8	15	4	5	40	4	47	4	48
Amino acids and their derivatives	2	22	8	2	8	24	0	24	7	4	3	23	23	6	25	8	13	6
Organic acids	6	7	1	14	7	14	2	20	2	7	3	27	16	5	20	1	26	1
Nucleotide and their derivatives	5	5	5	3	15	6	4	12	3	2	5	15	16	8	21	4	25	4
Alkaloids	6	5	7	0	7	5	2	4	3	1	3	6	14	5	12	3	16	4
Terpenoids	1	0	2	4	4	3	0	1	0	0	0	4	7	3	5	3	9	3
Lignans and coumarins	2	1	4	1	5	1	0	0	1	0	3	1	3	3	2	2	4	3
Tannins	0	2	1	0	1	2	1	2	0	1	1	1	0	2	0	3	0	1
Quinones	0	1	0	0	0	1	0	1	0	1	0	0	0	1	0	0	0	2
Steroids	1	0	0	2	1	0	0	1	0	0	0	1	2	0	2	0	2	0
Others	13	14	2	10	12	19	16	14	2	16	7	21	20	19	18	17	18	16
Total	91	86	68	86	112	116	66	114	31	54	57	131	202	127	217	119	217	119
	177		154		228		180		85		188		329		336		336	

The different metabolites were mapped to the KEGG pathways to investigate the involved biological processes (**Supplementary Table S2**). The top four KEGG pathways with the highest number of different metabolites in most pairwise comparisons were involved in “metabolic pathways”, “biosynthesis of secondary metabolites”, “biosynthesis of amino acids”, and “ABC transporters”, except for the H-S *vs*. H-R group (“biosynthesis of amino acids” was not on the top four lists of the KEGG pathways). In the roots, compared with CK, the most enriched KEGG pathways (*p*-value ≤ 0.01) common in L and H contained “biosynthesis of amino acids” and “biosynthesis of secondary metabolites”. In the shoots, compared with CK, the most enriched KEGG pathways (*p*-value ≤ 0.01) common in L and H contained “butanoate metabolism”. In the comparisons of S *vs*. R, only “phenylpropanoid biosynthesis” was significantly enriched (*p*-value ≤ 0.01) in H-S *vs*. H-R.

### 3.5 Profiles of primary metabolites under salinity

Lipids were identified as one of the most different primary metabolites in *S. europaea* seedlings under salt stress, and 60 and 26 different lipids were accumulated in the roots and shoots, respectively (**Supplementary Table S3**). With the increase of NaCl concentrations, 40 different lipids presented increasing accumulation levels in L-R (for example, lysoPE 15:1 was accumulated 81.9-fold higher in L-R than CK-R) and then rapidly decreased in H-R. The majority of these lipids were compounds of lysophosphatidylethanolamine (LPE) and lysophosphatidylcholine (LPC). Sixteen different lipids showed decreasing trends, and four different lipids exhibited an increasing accumulation. In the shoots, except for lysoPC 20:0 and lysoPC 17:1, the accumulation of 24 different lipids was reduced under the salinity treatments compared with the control, especially MAG(18:4) isomer3 and 2-linoleoylglycerol-diglucoside, which were not detected under the salinity treatments.

**Figure 5:**
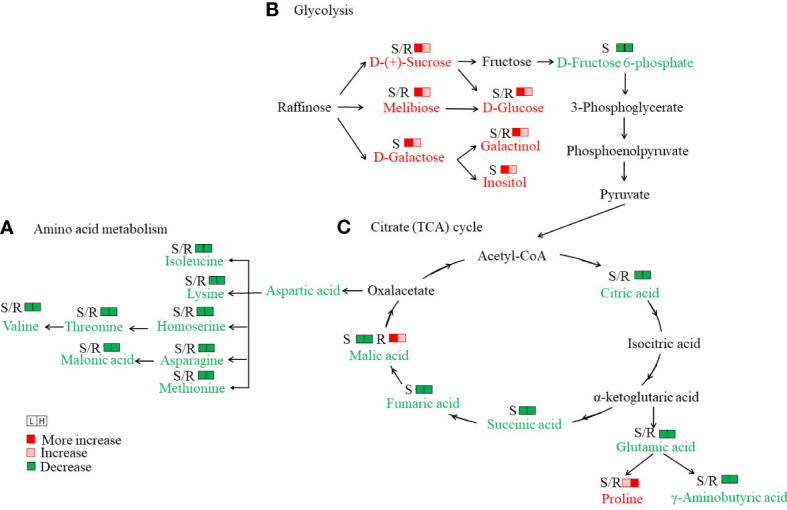
Analysis of important metabolic networks in the seedlings of *Salicornia europaea* under different NaCl conditions. **(A)** Amino acid metabolism, **(B)** Glycolysis, and **(C)** Citrate (TCA) cycle. S/R, shoot or root. The metabolites with a red color indicate an increase under both L (50 mM NaCl) and H (300 mM NaCl) conditions compared with CK (0 mM NaCl), while the metabolites with a green color indicate decreases under both L and H conditions compared with CK.

Amino acids and their derivatives were the second most abundant different primary metabolites in *S. europaea* seedlings under salt stress. In total, 35 and 32 different amino acids and their derivatives were detected in the roots and shoots, respectively (**Supplementary Table S3**). The different amino acids and their derivatives in the roots showed varied change patterns with increasing NaCl concentrations, including six members that increased continuously (the reduced form of glutathione was only detected in H-R), 10 members that evidently declined in L-R and then slightly increased in H-R, and 19 members that decreased significantly (alanylleucine was only detected in CK-R). To better understand the variation in individual amino acids, KEGG enrichment analysis was performed, and it was found that “biosynthesis of amino acids”, “lysine biosynthesis”, and “aminoacyl-tRNA biosynthesis” were the most enriched pathways in CK-R *vs*. L-R and CK-R *vs*. H-R. The amino acids related to these pathways declined drastically in both roots and shoots as NaCl increased (**Figure 5A**). In contrast, some amino acids such as L-proline and L-cysteine exhibited upward trends as the salinity increased. The amounts of L-proline and L-cysteine were increased by 1.46- and 2.11-fold under L-R compared with CK-R, respectively, and increased by 4.7- and 33.6-fold under H-R compared with CK-R, respectively. In the shoots, except for L-proline and L-cysteine which increased, other amino acids and their derivatives declined in L-S. Eleven members then increased slightly in H-S (only the contents of three members were higher in H-S than CK-S) and 20 members decreased continuously.

A total of 16 and 21 “saccharides and alcohols” were differently accumulated in the roots and shoots of *S. europaea* seedlings, respectively (**Supplementary Table S3**). Compared with CK, seven members showed a drastically high accumulation in L-R, H-R, and L-S, including D-glucose, sedoheptulose, isomaltulose, melibiose, D-(+)-sucrose, galactinol, and turanose. In particular, the content of turanose was 67.8- and 58.1-fold higher in L-R and H-R, respectively, than in CK-R. Compared with CK, D-(-)-arabinose accumulated significantly in L-R and L-S. In addition, the KEGG pathway of “galactose metabolism” was the most enriched in CK-S *vs*. L-S (**Supplementary Table S2**), and six metabolites were related to this pathway, including D-glucose, inositol, D-fructose 6-phosphate, melibiose, D-(+)-sucrose, and galactinol. Except for D-fructose 6-phosphate, the other metabolites were drastically elevated in L-S compared with CK-S (**Figure 5B**).

In total, 31 and 31 organic acids were differently accumulated in the roots and shoots of *S. europaea* seedlings, respectively (**Supplementary Table S3**). In the roots, four different change patterns emerged, including 12 members with increasing levels in L-R that then decreased in H-R (3-methyl-2-oxobutanoic acid was not detected in CK-R), 12 members that declined continuously, five members that gradually increased (2-furanoic acid was not detected in CK-R), and two members that declined in L-R and then slightly increased in H-R. In the shoots, the different organic acids also showed four different change patterns, in which 23 members declined gradually with increasing salinity. In particular, the KEGG pathway of “butanoate metabolism” was the most enriched (*p*-value ≤ 0.01) in CK-S *vs*. L-S, and the KEGG pathways of “butanoate metabolism” and “carbon metabolism” were the most enriched (*p*-value ≤ 0.01) in CK-S *vs*. H-S. The two pathways covered γ-aminobutyric acid, succinic acid, fumaric acid, L-(-)-malic acid, 3-hydroxypropanoic acid, citric acid, and gluconic acid. All these organic acids were decreased dramatically with increasing salinity compared with CK (**Figure 5C**).

### 3.6 Profiles of flavonoids under salinity treatments

Flavonoids are important secondary metabolites in plants adapted to salinity. A total of 34 and 39 different flavonoids were accumulated in the roots and shoots, respectively, under the three salinity conditions. Most of these flavonoids were involved in the pathway of “flavone and flavonol biosynthesis” (ko00944) based on the KEGG enrichment analysis. The different flavonoids are shown in the heat map in **Supplementary Figure S1**.

**Figure 6:**
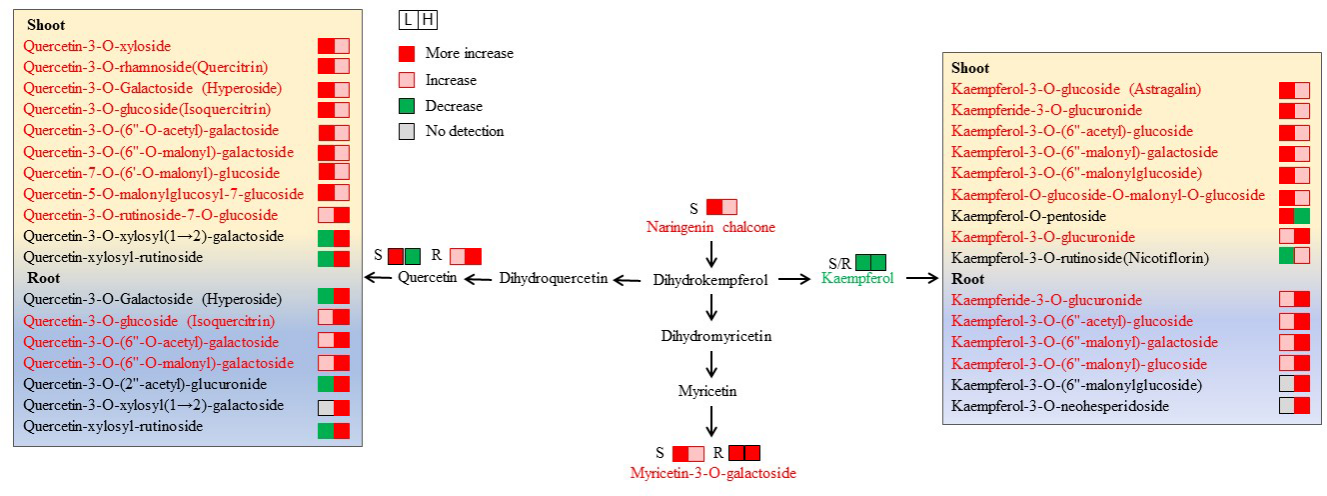
Changes in metabolites mapped to the metabolic pathway of flavone and flavonol biosynthesis. The metabolites with a red color indicate an increase under both L (50 mM NaCl) and H (300 mM NaCl) condition compared with CK (0 mM NaCl), while the metabolites with a green color indicate a decrease under both L and H condition compared with CK.

In the roots, the content of kaempferol decreased significantly upon salt application, becoming almost undetectable in L-R and H-R. However, some derivatives of kaempferol showed increasing accumulation levels and peaked in H-R, for example, kaempferol-3-O-(6″-malonyl)-galactoside and kaempferol-3-O-(6″-malonyl)-glucoside (**Figure 6**). In contrast, quercetin and some of its derivatives increased continuously under salinity and peaked at H-R, including quercetin-3-O-(6″-O-acetyl)-galactoside and quercetin-3-O-(6″-O-malonyl)-galactoside. Four derivatives of isorhamnetin exhibited a similar change trend with a significant decrease in L-R and an increase in H-R, especially isorhamnetin-O-glucoside-O-malonyl-O-glucoside, which was undetectable in L-R but highly accumulated in H-R (FC value = 13.6).

In the shoots, naringenin chalcone and nepetin showed significantly increased levels under increasing salinity and peaked at L-S, with FC values of 6.0 and 12.2 compared with CK-S, respectively. It was found that kaempferol declined evidently with the application of salt stress; however, six of the nine derivatives of kaempferol continued to accumulate and peaked at L-S (**Figure 6**). Moreover, quercetin and eight of 11 of its derivatives increased continuously under increasing salinity and peaked at L-S, and quercetin-3-O-rhamnoside (quercitrin) had the highest FC value (13.1) at L-S. Three anthocyanins were differentially accumulated in the shoots under different salt conditions. Among them, delphinidin-3-O-glucoside (mirtillin) and delphinidin-3-O-rutinoside increased constantly and peaked at H-S, while delphinidin-3-O-arabinoside decreased evidently.

### 3.7 Profiles of phenolic acids under salinity treatments

Phenolic acids were important different secondary metabolites in *S. europaea* seedlings under salt stresses. In total, 62 and 40 different phenolic acids were accumulated in the roots and shoots of *S. europaea* seedlings under salt stress, respectively. Most of these metabolites belonged to the derivatives of hydroxybenzoic acid and hydroxycinnamic acid and were involved in “phenylpropanoid biosynthesis” (ko00940). The accumulation patterns of the representative members are illustrated in the heat map in **Supplementary Figure S2**.

**Figure 7:**
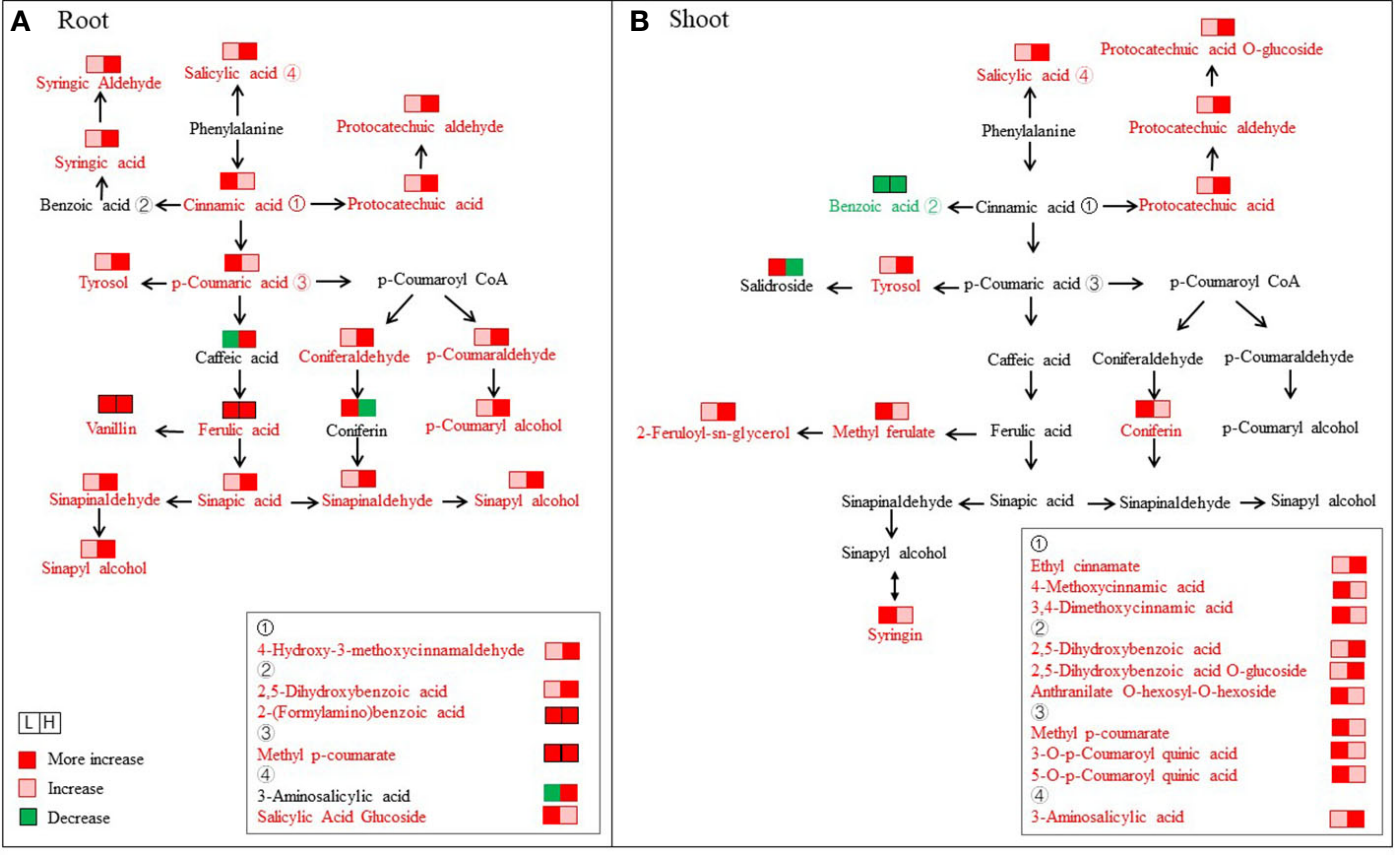
Changes in metabolites mapped to the metabolic pathway of phenylpropanoid biosynthesis. **(A)** Root. **(B)** Shoot. The metabolites shown in red color indicate increases under both L (50 mM NaCl) and H (300 mM NaCl) conditions compared with CK (0 mM NaCl), while the metabolites with a green color indicate a decrease under both L and H conditions compared with CK.

In the roots, some different phenolic acids presented significantly increasing trends with increasing salinity, including p-coumaraldehyde, p-coumaryl alcohol, coniferaldehyde, sinapyl alcohol, sinapinaldehyde, and 3-O-p-coumaroyl quinic acid (**Figure 7A**). In particular, the FC values of p-coumaraldehyde and p-coumaryl alcohol at H-R were 32.02- and 68.31-fold higher than at CK-R, respectively. A total of two hydroxybenzoic acids (protocatechuic acid and syringic acid) and five hydroxycinnamic acids (p-coumaric acid, caffeic acid, ferulic acid, sinapic acid, and chlorogenic acid) were detected with different change trends under varied NaCl conditions. Among them, protocatechuic acid, syringic acid, and sinapic acid showed continuously increasing levels with increasing salt concentrations. The contents of p-coumaric acid and ferulic acid under salinity treatments were significantly higher than CK-R, and peak values were found in L-R (FC values of 4.7 and 4.3, respectively). However, chlorogenic acid decreased quickly with increasing salinity.

In the shoots, we found the contents of some different phenolic acids increased under salinity and then peaked in L-S, including salidroside, coniferin, syringin, anthranilate O-hexosyl-O-hexoside, and so on, and their FC values were 2.3, 3.6, 5.0, and 9.6 than that in CK-S, respectively (**Figure 7B**). Differently, some different phenolic acids exhibited continuously increased levels with increasing salt conditions, including protocatechuic aldehyde, 2,5-dihydroxybenzoic acid, protocatechuic acid, salicylic acid, tyrosol, and protocatechuic acid among others.

### 3.8 Profiles of alkaloids under salinity

**Figure 8:**
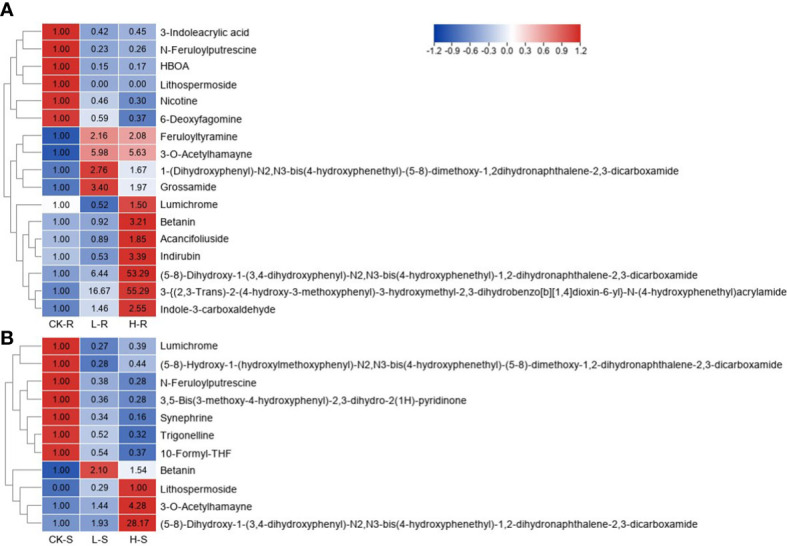
Heat map of different alkaloids among three different NaCl conditions. CK, 0 mM NaCl; L, 50 mM NaCl; H, 300 mM NaCl. **(A)** Heat map in the root (R) tissue. **(B)** Heat map in the shoot (S) tissue. Different metabolites were selected based on projection (VIP) >1 and fold change (FC) ≥2 or ≤0.5 in any of the comparison groups in S and R. The FC values are displayed in the heat map which uses the data of CK-R or CK-S as the calibrator, except for lithospermoside (no detection in the CK-S, and thus H-S is used as the calibrator).

In total, 17 and 11 different alkaloids were accumulated in the roots and shoots of *S. europaea* seedlings under salt stress. The change patterns of these alkaloids under different NaCl conditions are visualized in the heat map shown in **Figure 8**. In the roots, six different alkaloids significantly decreased after NaCl application compared with CK-R, while their contents in L-R and H-R did not differ greatly. In contrast, 11 different alkaloids showed increasing accumulation under salinity compared with CK-R. Among these alkaloids, some obtained maximum values in L-R, such as betanin, 3-O-acetylhamayne, and grossamide, while some members accumulated the highest levels in H-R, for example, the content of “(5-8)-dihydroxy-1-(3,4-dihydroxyphenyl)-N2,N3-bis(4-hydroxyphenethyl)-1,2-dihydronaphthalene-2,3-dicarboxamide” and “3-{(2,3-trans)-2-(4-hydroxy-3-methoxyphenyl)-3-hydroxymethyl-2,3-dihydrobenzo[b][1,4]dioxin-6-yl}-N-(4-hydroxyphenethyl)acrylamide” was 55.3- and 53.3-fold higher in H-R compared with CK-R, respectively. In the shoots, there were two different change trends of the different alkaloids, except for betanin and lithospermoside. Seven members decreased significantly under increasing NaCl conditions, the contents of which differed little between L-S and H-S, such as synephrine and lumichrome. Three members showed increasing trends with increasing NaCl concentrations, especially “(5-8)-dihydroxy-1-(3,4-dihydroxyphenyl)-N2,N3-bis(4-hydroxyphenethyl)-1,2-dihydronaphthalene-2,3-dicarboxamide” (the FC value in H-S was 28.2).

## 4 Discussion

### 4.1 *S. europaea* is suitable for studying metabolic change responses to salt stress

Salinity is a major abiotic stress affecting the productivity of important economic crops worldwide (Negrão et al., 2017). Under salt stress, the comprehensive metabolic responses of halophytes are altered. This metabolic reprogramming offers an opportunity to identify important salt-responsive compounds (Wang et al., 2019b). In the present study, under the application of different NaCl concentrations, *S. europaea* exhibited excellent performance accompanied by increased tissue succulence and relatively stable K^+^ levels, providing a model halophyte for salt tolerance research. Stress defense and active growth suppression are two complementary strategies for plants in adverse environments (Zhang et al., 2020b). *S. europaea* was able to survive under a very high NaCl concentration (800 mM) accompanied by evident growth inhibition (**Figure 1**), indicating that *S. europaea* positively regulated survival under high salinity by actively suppressing growth and resetting the balance between salt resistance and growth. The mechanism of this beneficial survival strategy has yet to be defined. In addition, in an intriguing twist, the results showed that lower NaCl concentrations were beneficial for *S. europaea* to promote growth, while higher NaCl concentrations directly inhibited growth. However, the diversity of metabolites and their functional characterization among low- and high-NaCl treatments have not yet been compared in *S. europaea*. In the present investigation, an abundant and diverse set of metabolites was identified in the roots and shoots of *S. europaea* exposed to various NaCl conditions, including primary metabolites and secondary metabolites.

### 4.2 Lipid composition is involved in the salt resistance of *S. europaea*

Salt stress often impairs the plasma membrane, and thus changes in membrane composition and properties are crucial in imparting salt tolerance among several higher plants (Mansour et al., 2015). Lipids are important in maintaining the stability and fluidity of the membrane, and regulatory roles are affected by changes in lipid composition (Gogna et al., 2020). Previous studies reported that the maintenance of membrane fluidity was regulated by the levels of unsaturated fatty acids (Kerkeb et al., 2001; Turk et al., 2004). In the roots of *S. europaea* subjected to salt stress, two unsaturated fatty acids namely 9,12,13-trihyroxy-10,15-octadecadienoic acid and 9,10,13-trihyroxy-11-octadecadienoic acid increased significantly. The major compounds of LPE and LPC were evidently accumulated in L-R. LPE and LPC are involved in the acyl modification of fatty acid unsaturation, and LPE may protect membrane integrity by inhibiting membrane lipid degradation (Ryu et al., 1997). Hence, the increased content of important unsaturated fatty acids and the regulation of LPE and LPC might be involved in the salt resistance of *S. europaea*. In addition, different variations were detected in lipids in the roots and shoots exposed to salinity; unlike lysoPC 20:0 and lysoPC 17:1, which increased significantly, the other different lipids decreased in the shoots under salt stress, implying that major changes in lipid composition might not be necessary to maintain plasma membrane fluidity in the shoots. It was concluded that lipid composition varied in different tissues, which enabled *S. europaea* to adapt so successfully to external NaCl treatments.

### 4.3 Salinity stress disturbs another type of primary metabolism in *S. europaea*

Plant metabolism is significantly perturbed under abiotic stress (Sanchez et al., 2008; Kumari et al., 2015). In the present study, with the exception of lipids, many different primary metabolites were found as belonging to the classes of “amino acids and their derivatives”, “saccharides and alcohols”, and organic acids. It is well known that amino acids and their derivatives regulate plant defense against abiotic stress by maintaining the osmotic balance and the stability of the cell membrane (Khan et al., 2019). The previous studies of Shelden et al. (2016) and Li and Song (2019) revealed a significant increase in certain amino acids (*i*.*e*., proline) under salt stress in barley and *S. salsa*. In accordance with these previous findings, in the present study, it was found that L-proline and L-cysteine accumulated continuously with increasing NaCl conditions both in the roots and shoots of *S. europaea*. As an osmotic regulator substance, proline enhances cell tolerance and reduces cell damage from various abiotic stresses (Zhao et al., 2021). Thus, proline may be an important osmotic regulator in *S. europaea* subjected to salt stress. In contrast, the levels of some amino acids in both the roots and shoots decreased significantly under NaCl treatments compared with the control (**Figure 5A**), and the KEGG pathways of “lysine biosynthesis” and “aminoacyl-tRNA biosynthesis” were highly enriched compared with the other pathways. Studies have shown that amino acids can function as the precursors of secondary metabolites that can protect plants from various stresses (Zhang et al., 2018). Thus, it was concluded that these decreased amino acids might be degraded or participate in the synthesis of other metabolites for regulating amino acid metabolism and deploying defense responses to salinity stress.

Saccharides can function as compatible osmolytes for osmotic adjustment and detoxification (Li and Song, 2019). As a primary adaptive strategy in response to stress, the biosynthesis and accumulation of some soluble sugars are often induced under salinity (Jia et al., 2019). Wang et al. (2020) stated that some soluble sugars including glucosamine, maltose, and D-(+)-sucrose increased significantly in the leaves of *S. salsa* under salt stress. A high accumulation of fructose and glucose in the halophyte *Limonium albuferae* was found under high-salinity conditions, and they were considered as major osmolytes in *Limonium* genus (González-Orenga et al., 2019). Similarly, our data showed that salt treatment upregulated the sucrose and glucose contents, indicating that they might be the main osmotic adjustment substances involved in *S. europaea*’s resistance to salt stress. In addition, it was found that the saccharide metabolites associated with glycolysis increased significantly under salinity under L-S compared with CK (**Figure 5B**). As an important process of energy production, the increase of glycolysis metabolites could be linked to the enhanced production of reactive oxygen species (ROS) (Guo et al., 2022). Thus, an elevated accumulation of glycolysis metabolites might be coupled with the generation of ROS in *S. europaea* especially.

Organic acids are osmotic regulator substances in plants and play important roles in the physiological process of salt resistance (Sanchez et al., 2008). In the present study, it was found that salinity induced the increase of certain kinds of organic acids in both the roots and shoots of *S. europaea*, especially 3-methyl-2-oxobutanoic acid, trans,trans-muconic acid, and 2,3-dihydroxybenzoic acid (**Supplementary Table S3**). Hence, these organic acids might accumulate more contents to decrease the osmotic potential and help *S. europaea* deal with salt stress. In addition, the citrate (TCA) cycle is an important pathway to release more energy and accelerate the physiological metabolic reaction against stress (Zhang et al., 2016). A detailed analysis showed that some organic acids that were TCA cycle intermediates decreased to a marked degree in the shoots of *S. europaea*, and their contents were lower in H than in L (**Figure 5C**). Similar observations have been reported in the halophytes *Thellungiella halophila* and *Limonium latifolium* in response to salt stress, suggesting that many changes in organic solute composition are predominantly controlled by constitutive developmental programs (Gong et al., 2005; Gagneul et al., 2007). The decrease of these metabolites might be accompanied by the suppression of energy production, implying that *S. europaea* adopted an energy conservation strategy from plant growth to the induction of protective mechanisms under salt stress; meanwhile, the inhibited TCA cycle flux might partially counteract ROS overproduction associated with glycolysis as a response of *S. europaea* to salt (Guo et al., 2022).

### 4.4 Flavonoids and phenolic acids positively dominate the salt resistance of *S. europaea*

When plants are exposed to a saline environment, ROS overgeneration disrupts the balance between ROS production and scavenging, inducing the oxidative damage of cells (Hao et al., 2021). Halophytes evolve a multifaceted antioxidant defense network to reduce ROS overgeneration under abiotic stress (Şirin and Aslım, 2019; Li et al., 2022). The phenylpropanoid pathway involves the biogenesis of diverse phenolic polymers, such as flavonoids and phenolic acids, which play important roles of ROS scavenging in plant defense against abiotic stresses (Liu et al., 2020; Li et al., 2021a). In the present study, KEGG analyses revealed that the pathways of “phenylpropanoid biosynthesis” and “flavone and flavonol biosynthesis” were dramatically enriched. Our results were consistent with previous reports (Li and Song, 2019; Wang et al., 2019a). For example, in peanut response to salt stress, “phenylpropanoid biosynthesis” and “flavonoids biosynthesis” were also enriched, and a dramatic accumulation of flavonoids and phenolic compounds was found, revealing that the abundances of these metabolites were regulated by salinity or salinity recovery specifically (Cui et al., 2018).

Flavonoids are well-known secondary metabolites produced when plants encounter abiotic stress, including flavone, flavonol, and flavonoid (Górniak et al., 2019; Wang et al., 2019a). Flavonoids occur in plants as glycosides and commonly function by increasing the water solubility to protect their reactive groups from oxidation by free radicals (Agati and Tattini, 2010). In addition, glycosylated flavonoids can be further acylated by aliphatic or aromatic acetyl groups (Zhang et al., 2018). In the current study, glycosylated flavonoids, malonylated flavonoids, and flavonoids with acetyl modifications were found in the seedlings of *S. europaea* (**Supplementary Figure S1**). In particular, it was found that these flavonoids displayed distinct variation patterns under L and H and in both the shoots and roots (**Figure 6**), implying that flavonoids with different substituent groups might play different roles in different tissues of *S. europaea* in response to salinity. Furthermore, flavonoids with different chemical structures possess different levels of antioxidant activity. Researchers have reported that ortho-dihydroxylated B-ring flavonoids increase in plants under stress and function as important potential ROS scavengers, including quercetin and its derivatives (Hernández et al., 2009). Quercetin is an anti-oxidative flavonoid with a wide distribution throughout plants and serves as a promising candidate for cancer prevention (Qiu et al., 2010; Li et al., 2019). Kaempferol-3-O-glucoside is a bioactive flavonoid with antioxidant, anti-inflammatory, and protective characteristics (Kashyap et al., 2017). The significantly increased kaempferol derivatives, quercetin, and quercetin derivatives detected in the present study might scavenge the ROS in the shoots and roots to protect the plant from oxidative stress caused by increased salinity. Interestingly, most of these compounds were highly accumulated under low NaCl conditions in the shoots, while they were highly accumulated under high NaCl conditions in the roots. The results indicated that coping strategies in the shoots and roots under increasing salinity varied by modulating flavonoid accumulation with different substituent groups. However, the exact roles that these compounds play in salinity stress response remain to be investigated.

As another major category with antioxidant properties, phenolic acids are generally distributed in plant cell walls (Pradeep and Sreerama, 2018; Li et al., 2021b). In the present study, it was found that some of the phenolic acids were significantly higher in the seedlings of *S. europaea* both in the shoots and roots under the NaCl treatments, and the contents of these phenolic acids increased consistently with increased NaCl concentrations, including protocatechuic acid, protocatechuic aldehyde, salicylic acid, and tyrosol (**Figure 7**, **S2**). Among them, protocatechuic acid is a natural phenolic acid with biologically active components such as anti-aging, anti-atherosclerotic, and antibacterial activities (Wang et al., 2020). Similarly, as a plant endogenous signal molecule, salicylic acid possesses the ability to improve the metabolic activities and reduce the inhibition effects of plant growth under salinity (Bai et al., 2009). Changes in the level of salicylic acid were observed in the salt-tolerant wild soybean compared with the common wild soybean (Jiao et al., 2018). Besides, *p*-coumaric acid is a chain-breaking antioxidant by radical scavenging activity related to its electron- or hydrogen-donating ability and to the capacity to delocalize/stabilize the resulting phenoxyl radical within its structure (Pei et al., 2016). It was also found that the content of *p*-coumaric acid increased significantly in the roots of *S. europaea* under salt stress. We speculated that the increased contents of these phenolic acids were relevant to the antioxidant content and antioxidant activity, protecting *S. europaea* against oxidative stress due to salinity. Interestingly, the relative contents of some phenolic acids in the shoots and roots were significantly different due to different NaCl treatments (**Figure 7**). Generally, for the phenolic acids participating in the process of phenylpropanoid biosynthesis, most were highly accumulated in the roots and increased with increasing NaCl concentration. Only a small number of phenolic acids increased significantly downstream of the pathway, including syringin, coniferin, and tyrosol. Plant root systems are exposed directly to saline environments and must regulate metabolic processes firstly to maintain life activities (Li et al., 2017). It was speculated that these upregulated phenolic acids in the process of phenylpropanoid biosynthesis reflected the different defense effects of the roots compared with the shoots. Furthermore, suberin is a complex polyester of aliphatics and phenolics deposited in the cell walls that creates a hydrophobic barrier to control the movements of water and solutes through cell walls, thus playing important roles in the salt adaptation of halophytes (Cui et al., 2021). Coumaric, caffeic, and ferulic acids are revolved in the suberin biosynthetic pathway, and they are linked to fatty alcohols by BAHD-type acyltransferases to produce alkyl hydroxycinnamates found in suberin-associated waxes (Vishwanath et al., 2015). Thus, the significant accumulation of coumaric, caffeic, and ferulic acids in the roots might contribute to suberin biosynthesis in *S. europaea*, thereby further helping to reduce the uncontrolled transport of water and dissolved ions (*e*.*g*., excess Na^+^) through the apoplast and transcellularly.

As a class of basic organic compounds in nature, alkaloids exhibit strong antioxidant and anti-tumor activities (Li and Song, 2019). In the present study, some alkaloids were significantly upregulated in the seedlings of *S. europaea*, including betanin, 3-O-acetylhamayne, and “(5-8)-dihydroxy-1-(3,4-dihydroxyphenyl)-N2,N3-bis(4-hydroxyphenethyl)-1,2-dihydronaphthalene-2,3-dicarboxamide” (**Figure 8**). The results implied that alkaloids might also play key roles in *S. europaea* under salt stress, which needs to be further validated.

## 5 Conclusion

The present study analyzed the physiological changes of *S. europaea* under different NaCl concentrations and unraveled the metabolic differences using widely targeted metabolomic analysis. *S. europaea* exhibited excellent performance under a variety of NaCl conditions, and moderate NaCl concentrations could promote the growth of the plant. The current investigation revealed that the biosynthesis of some key primary metabolites, including lipids, amino acids, soluble sugars and organic acids, was significantly enhanced under NaCl treatment. The significant changes in the contents of secondary metabolites corresponded well to the changes of saccharides, flavonoids, and phenolics acids, which were highly associated with the glycolysis pathway, phenylpropanoid biosynthesis, and flavonoid biosynthesis. These results provide new perspectives on important metabolites and their crucial contributions to the salt tolerance of *S. europaea*.

## Data availability statement

The original contributions presented in the study are included in the article/**Supplementary Material**. Further inquiries can be directed to the corresponding authors.

## Author contributions

GC and HY designed and supervised the project. HD performed bioinformatic analysis and drafted the manuscript. RT and FT performed the experiments and participated in the bioinformatic analysis. YL and QZ collected the samples, maintained the growth of the experiment materials, and measured the physiological values. YH revised the manuscript and participated in the interpretation of the data. GC and HY contributed equally as corresponding authors. All authors contributed to the article and approved the submitted version.

## Supplementary material

Supplementary Figure 1 Heat map of different metabolites of flavonoids among three different NaCl conditions. CK, 0 mM NaCl; L, 50 mM NaCl; H, 300 mM NaCl. **(A)** Heat map in the root (R) tissue. **(B)** Heat map in the shoot (S) tissue. Different metabolites were selected based on projection (VIP) >1 and fold change (FC) ≥2 or ≤0.5 in any of the comparison groups in S and R. The FC values are displayed in the heat map which uses the data of CK-R or CK-S as the calibrator, except for kaempferide-3-O-glucuronide and kaempferol-3-O-(6″-malonylglucoside) (no detection in the CK-R, and thus H-R is used as the calibrator).

Supplementary Figure 2 Heat map of different phenolic acids among three different NaCl conditions. CK, 0 mM NaCl; L, 50 mM NaCl; H, 300 mM NaCl. **(A)** Heat map in the root (R) tissue. **(B)** Heat map in the shoot (S) tissue. Different metabolites were selected based on projection (VIP) >1 and fold change (FC) ≥2 or ≤0.5 in any of the comparison groups in S and R. The FC values are displayed in the heat map which uses the data of CK-R or CK-S as the calibrator, except for terephthalic acid, pyrocatechol (no detection in the CK-R, and thus H-R is used as the calibrator), and methyl p-coumarate (H-S is used as the calibrator).

Supplementary Table 1 Metabolites detected in *Salicornia europaea* seedlings.

Supplementary Table 2 Kyoto Encyclopedia of Genes and Genomes pathways of different metabolites among different comparison groups.

Supplementary Table 3 Classification of primary metabolites detected in the roots and shoots of *Salicornia europaea* seedlings under salt stress.
