# Supplementary figures and images for: Metabolomics analysis unveils important changes involved in the salt tolerance of Salicornia europaea
Source: Front Plant Sci. 2023 Jan 20;13:1097076. doi: 10.3389/fpls.2022.1097076 (PMC9896792; doi:10.3389/fpls.2022.1097076)

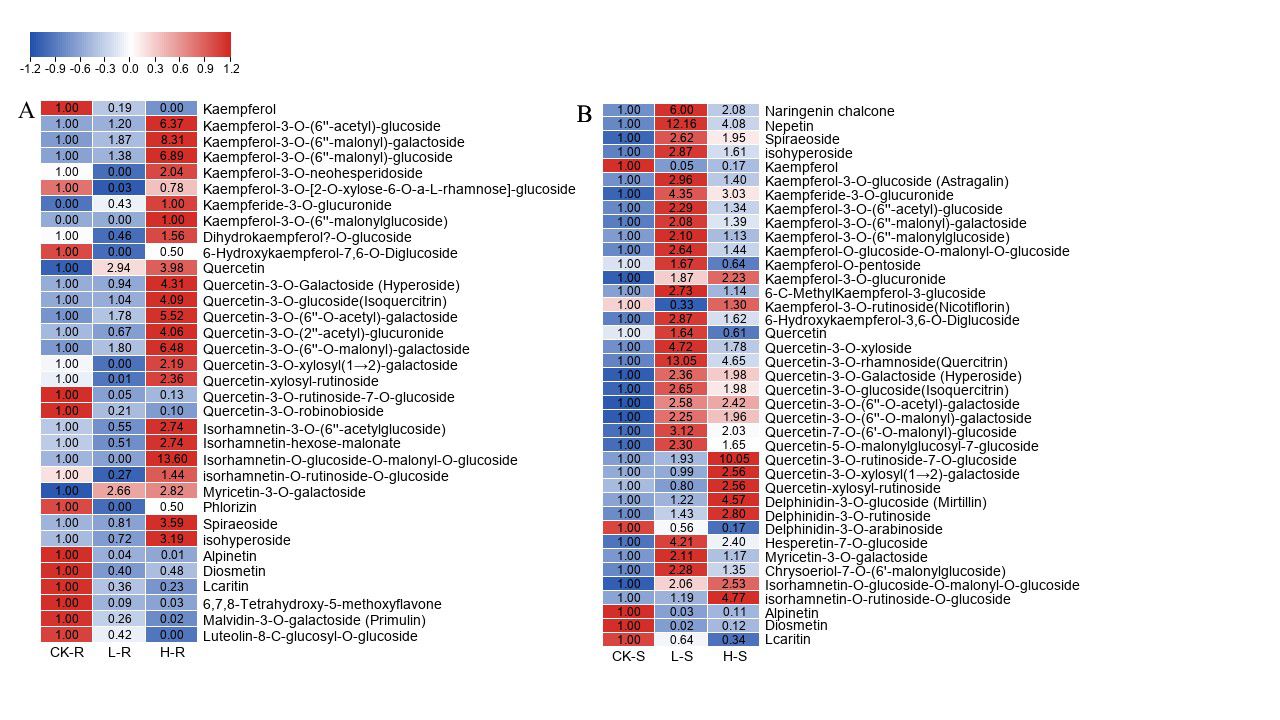

Supplement: Supplementary Figure 1 — Heat map of different metabolites of flavonoids among three different NaCl conditions. CK, 0 mM NaCl; L, 50 mM NaCl; H, 300 mM NaCl. (A) Heat map in the root (R) tissue. (B) Heat map in the shoot (S) tissue. Different metabolites were selected based on projection (VIP) >1 and fold change (FC) ≥2 or ≤0.5 in any of the comparison groups in S and R. The FC values are displayed in the heat map which uses the data of CK-R or CK-S as the calibrator, except for kaempferide-3-O-glucuronide and kaempferol-3-O-(6″-malonylglucoside) (no detection in the CK-R, and thus H-R is used as the calibrator). [file Image_1.jpg]

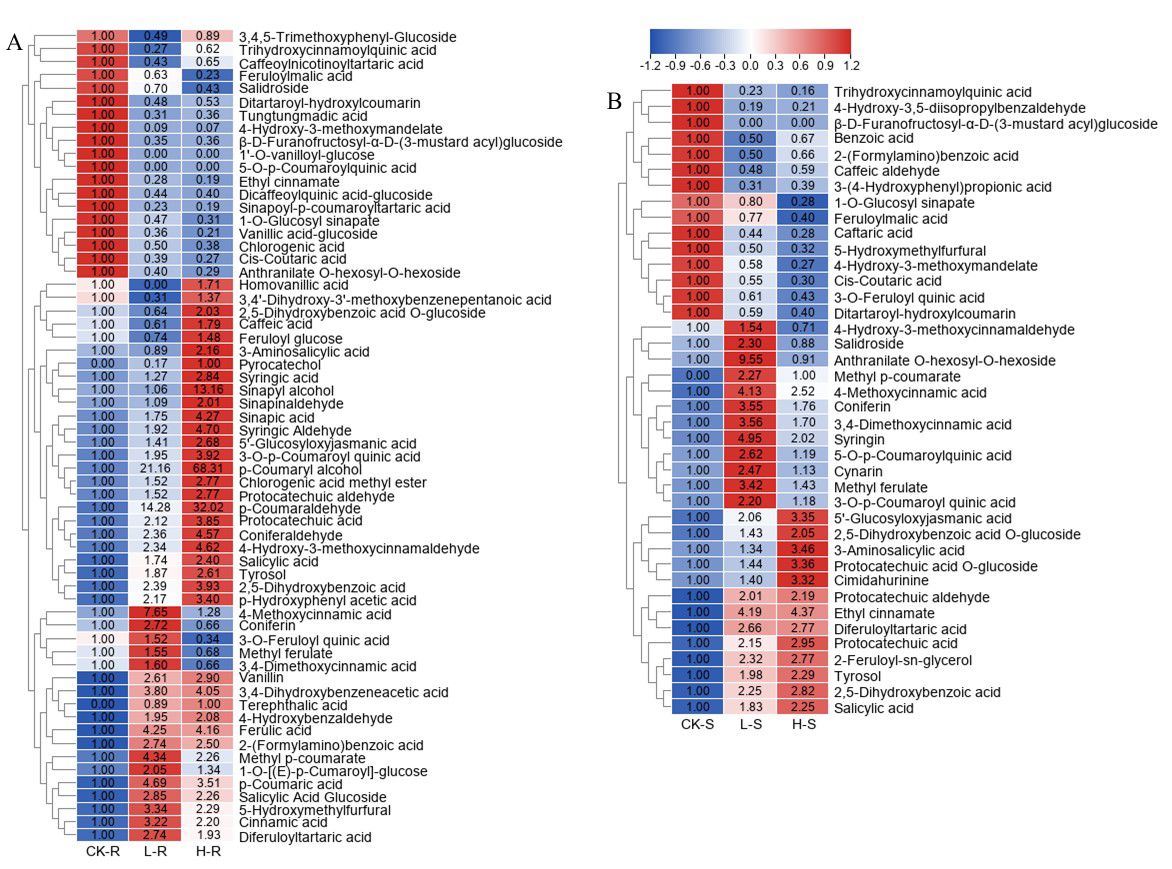

Supplement: Supplementary Figure 2 — Heat map of different phenolic acids among three different NaCl conditions. CK, 0 mM NaCl; L, 50 mM NaCl; H, 300 mM NaCl. (A) Heat map in the root (R) tissue. (B) Heat map in the shoot (S) tissue. Different metabolites were selected based on projection (VIP) >1 and fold change (FC) ≥2 or ≤0.5 in any of the comparison groups in S and R. The FC values are displayed in the heat map which uses the data of CK-R or CK-S as the calibrator, except for terephthalic acid, pyrocatechol (no detection in the CK-R, and thus H-R is used as the calibrator), and methyl p-coumarate (H-S is used as the calibrator). [file Image_2.jpg]
